# Effect of surgeon on transprosthetic gradients after aortic valve replacement with Freestyle^® ^stentless bioprosthesis and its consequences: A follow-up study in 587 patients

**DOI:** 10.1186/1749-8090-2-40

**Published:** 2007-10-05

**Authors:** Alexander Albert, Ines Florath, Ulrich Rosendahl, Wael Hassanein, Eberhard V Hodenberg, Stefan Bauer, Ina Ennker, Jürgen Ennker

**Affiliations:** 1 Department of cardiac surgery, Heart Institute Lahr/Baden, Hohbergweg 2, 77933 Lahr/Germany; 2 Department of cardiology, Heart Institute Lahr/Baden, Hohbergweg 2, 77933 Lahr/Germany

## Abstract

**Background:**

The implantation of stentless valves is technically demanding and the outcome may depend on the performance of surgeons. We studied systematically the role of surgeons and other possible determinants for mid-term survival, postoperative gradients and Quality of Life (QoL) after aortic valve replacement (AVR) with Freestyle^® ^stentless bioprostheses.

**Methods:**

Between 1996 and 2003, 587 patients (mean 75 years) underwent AVR with stentless Medtronic Freestyle^® ^bioprostheses. Follow-up was 99% complete. Determinants of morbidity, mortality, survival time and QoL were evaluated by multiple, time-related, regression analysis. Risk models were built for all sections of the Nottingham Health Profile (NHP): energy, pain, emotional reaction, sleep, social isolation and physical mobility

**Results:**

Actuarial freedom from aortic valve re-operation, structural valve deterioration, non-structural valve dysfunction, prosthetic valve endocarditis and thromboembolic events at 6 years were 95.9 ± 2.1%, 100%, 98.7 ± 0.5%, 97.0 ± 1.5%, 79.6 ± 4.3%, respectively. The actuarial freedom from bleeding events at 6 years was 93.1 ± 1.9%. Estimated survival at 6 years was similar to the age-matched German population (61.4 ± 3.8 %). Predictors of survival time were: diabetes mellitus, atrial fibrillation, peripheral vascular disease, renal dysfunction, female gender > 80 years and patients < 165 cm with BMI < 24. Predictive models showed characteristic profiles and good discriminative powers (c-indexes > 0.7) for each of the 6 QoL sections. Early transvalvular gradients were identified as independent risk factors for impaired physical mobility (c-index 0.77, p < 0.002). A saturated propensity score identified besides patient related factors (e.g. preoperative gradients, ejection fraction, haematological factors) indexed geometric orifice area, subcoronary implantation technique and individual surgeons as predictors of high gradients.

**Conclusion:**

In addition to the valve size (in relation to body size), subcoronary technique (versus total root) and various patient-related factors the risk of elevated gradients after stentless valve implantation depends, considerably on the individual surgeon.

Although there was no effect on survival time and most aspects of QoL, higher postoperative transvalvular gradients affect physical mobility after AVR.

## Background

Stented bioprostheses are considered at risk of structural failure and a non physiological flow pattern. Since the rigid stent is considered to be incremented in these disadvantages, stentless bioprostheses were developed. During the last years stentless bioprostheses have been used frequently and clinical outcome has been demonstrated [1-3].

The implantation of stentless valves is considered as technically more demanding. Nevertheless no increase in peri-operative risk in comparison to the implantation of stented bioprostheses or mechanical valves was observed in our own [4] and the experience of others [5-8] and favourable hemodynamic performance of the stentless valves has been demonstrated [1,2,9-11]. However, different groups have observed a heterogeneity in transvalvular Doppler gradients early after implantation and some patients have been found to have higher transvalvular gradients than anticipated [1,12]. It is still unclear to what extent patient related factors (e.g. preoperative gradient), characteristics of the early postoperative period like elevated stroke volume, local oedema and hematoma or other surgical factors are associated with this phenomena. [13]; however it was assumed that elevated postoperative gradients observed in stentless valves depend to a larger extent on the surgeon's skill and experience [1,12]. It is also a matter of debate if higher gradients early after surgery are just a transient phenomena or are persistent over time, affecting clinical outcome [1,12-14].

The purpose of the present study is to estimate the clinical importance of the individual surgeons for quality of life (QoL) and survival after aortic valve replacement (AVR) with Freestyle^® ^stentless bioprostheses. Thereby early postoperative gradients, the relationship between prosthesis-size and patient size, the implantation technique (subcoronary versus total root) and a wide spectrum of patient's characteristics including online accessible laboratory values were used as risk-adjustment variables in multivariate analysis.

## Methods

### Patient Population

Between April 1996 and December 2003, 587 patients older than 60 years underwent AVR with the stentless biological Medtronic Freestyle^® ^Prosthesis (for age distribution see additional file 1). This group of patients represents 31 % of all patients receiving aortic valve prosthesis in this age group in our centre. The choice of the prosthesis type was according to surgeon's preference and patient's choice after informed consent. Additional files 2&3 show the operative and preoperative characteristics of the patient population. All patients have signed an informed consent for the operation, for quality control measures and the follow-up studies.

The indexed geometric orifice area (IGOA) were calculated by the internal diameters for corresponding valve sizes reported by the company [Data from Medtronic: Valve size 19, 21, 23, 25, 27 – Internal Diameters 16, 18, 20, 21.5, 23.5] divided by BSA.

### Technique of implantation and the individual surgeons

All operations were performed using standard cardio-pulmonary bypass techniques with systemic normothermia and both antegrade and retrograde hyperkalemic cold blood cardioplegia. The subcoronary implantation was performed using a 4/0 Prolene continuous running suture for both, the first and second suture line. The prosthesis usually was 120° rotated in order to place the Dacron covered muscular part of the implant towards the human non-coronary sinus. Total root implantation was performed using a 4/0 Prolene continuous running suture for the annular implantation and distal anastomoses to the aorta and a 5/0 Prolene suture for coronary ostia re-implantation. The individual surgeons were included into the multivariate models as nominal data. The majority of the stentless valves were implanted by 5 surgeons (surgeons A-E), one surgeon has implanted only 28 valves (surgeon F); a group of younger surgeons, who operated a total of 15 valves, were subsumed as a mixed group M.

### Echocardiography

Intraoperative transesophageal echocardiography and transthoracic echocardiographic control before hospital discharge (4 to 7 days after AVR) under rest were performed by experienced cardiologists. Gradients from the pre-discharge echocardiography were used as variable for the present study. Mean transvalvular gradient was calculated by continuous wave Doppler using the modified Bernoulli equation. In cases with unusual higher gradients or other irregular findings a second opinion by a colleague was asked for.

### Follow-up

Follow-up information was obtained 6 month after surgery, in 2000, 2001 and 2003 by mailed questionnaires and completed by telephone interviews. Follow-up was 99% complete. Mean follow-up time was 32 ± 23 months. The follow-up questionnaire consisted of a quality of life (QoL) assessing questionnaire, the Nottingham Health Profile (NHP) [15], and general questions concerning postoperative complications, further hospitalisation and NYHA status.

### Statistical Analysis

Statistical analysis was performed using the software package SPSS (SPSS Inc, Chicago, IL).

For each patient included in the present study, 49 pre-operative characteristics were retrieved from the clinical information system and the consolidated database of our Data Mart system [16]. The data is based on continuous, online input from the anaesthesiological, cardio-surgical quality assurance and the laboratory data of the clinical chemistry [Tables a1, a2, additional files].

#### Valve-related morbidity was estimated by life-table analysis

By logistic regression the following categorical outcome variables were analysed: 30-days- and 6-months-mortality and QoL. NHP-scores above the values of the general German population of men and women of the same age [17] were defined as an impaired QoL and were coded by 1. We did not analyze the QoL as continuous data, because the NHP-scores were not normally distributed. In order to assess the effect of missing data in the models for Quality of Life, we have recalculated all models. Firstly, we inputted all missing data as "impaired" (worst case); secondly, we inputted all missing data as "non impaired" (best case).

#### Predictors for survival time were identified by Cox-regression analysis

For variable selection of all multivariate regression models, the Akaike Information Criterion (AIC = Deviance of the model + 2 * number of included parameters) was calculated for variables showing a difference for the outcome variable with a p value smaller than or equal to 0.25. The variables were included into multiple regression models in a stepwise way. The AIC was calculated each time a variable was included. The final model was reached when no more reduction in AIC was observed. To establish linear dependency of outcome from continuous variables, patient population was divided into subgroups of the same size. The logit of the outcome variable was calculated within each group. In the case of a nonlinear increase or decrease of an event, the AIC of the models was determined for several cut points or quadratic relations.

For Cox-analysis the different Kaplan-Meier curves were inspected after dividing the patient population in equally sized subgroups. The relevant cut points were then determined by minimization of the AIC of the Cox-model.

## Results

**Figure 1:**
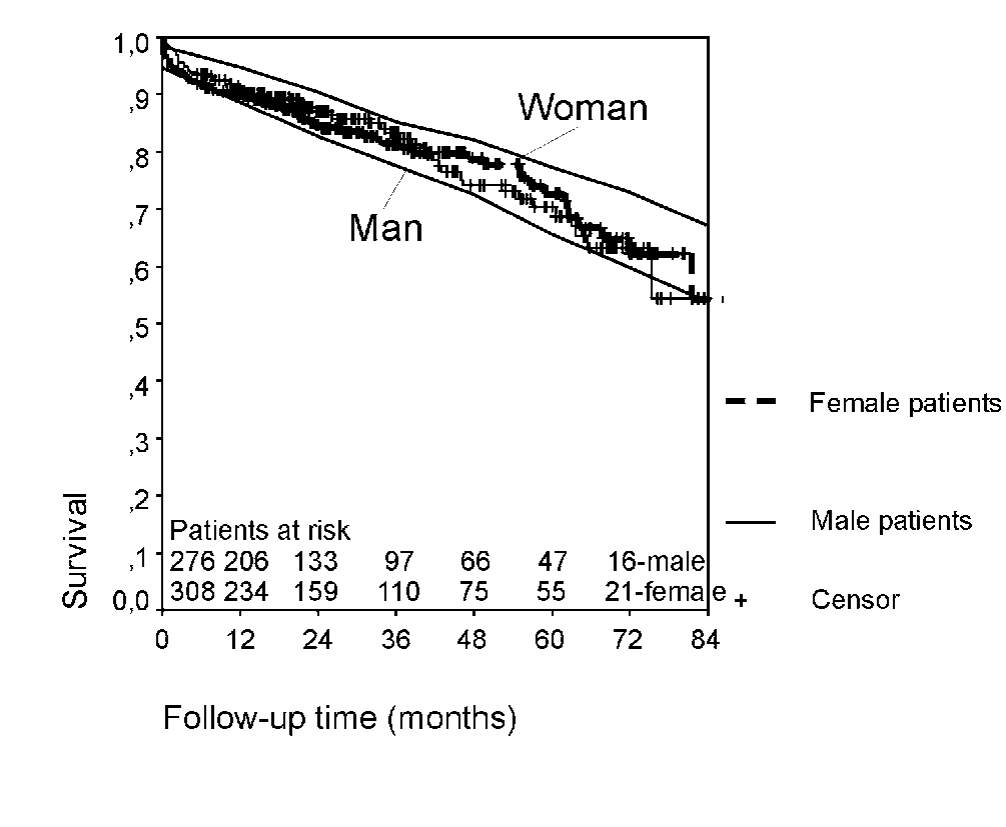
**Survivor functions for patients after AVR compared to the age- and gender matched German population**. The actuarial survival rate at 6 years was 61.4 ± 3.8 % and comparable to the estimated survival function for 75 year-old male and female Germans.

The postoperative mortality after 1 and 6 months was 3.1% (18 patients) and 7.3 % (43 patients) respectively. During follow-up time up to 86 months, 97 patients of the hospital survivors (569 patients) died. The actuarial survival rate at 6 years was 61.4 ± 3.8 % and comparable to the estimated survival function for 75 year-old male and female Germans (Fig. 1).

76% of all survivors (356 patients) responded to the mailed questionnaire, assessing NYHA-status and QoL and those who did not respond were contacted by telephone. Of all non-responders (N = 113) the reasons for non-responding were non-cardiac-illness (8%), cardiac illness (19%), disagreement (34%), staying abroad (2%) and without reason (willingly answered on the phone, however, did not return the questionnaire) (38%).

**Figure 2:**
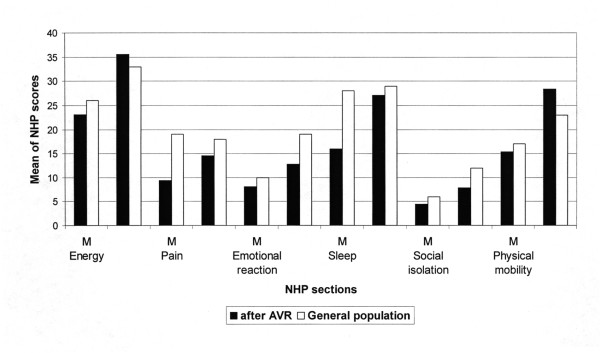
**Mean scores of the six NHP sections compared to the age- and gender-matched general German population**. The QoL after AVR with the stentless bioprosthesis over the follow-up time was compared to the general German population of the same age and gender. Besides from the section "pain" the patients after AVR showed no significant differences to the normal population. Our observation that the patients after AVR have lower values for pain may be explained by misunderstanding of the questionnaires: the patients thought that the question asking for pain mean only cardiac related pain, whereas in the general population all other kinds of pain were included.

The QoL after AVR with the stentless bioprosthesis over the follow-up time was compared to the general German population of the same age and gender [17] (figure 2). Apart from pain, which showed significantly lower values, all sections had normal values after AVR.

### Valve-related morbidity

Actuarial freedom from aortic valve re-operation, structural valve deterioration, non-structural valve dysfunction, prosthetic valve endocarditis and thrombembolic events at 6 years were 95.9 ± 2.1%, 100%, 98.7 ± 0.5%, 97.0 ± 1.5%, 79.6 ± 4.3%, respectively. In 1389 patient-years, twelve patients had to be re-operated (0.9%/pt-yr). The indications to re-operation were mainly due to outflow obstruction. No significant aortic insufficiency was observed (additional file 4). The quality of life after re-replacement of aortic valve was impaired for the sections energy, pain, sleep and physical mobility.

80 (16.8%) patients required anticoagulation therapy due to co-morbidities and 15 major bleeding events were observed (1.1%/pt-yr). The actuarial freedom from bleeding events at 6 years was 93.1 ± 1.9%.

### Predictors of valve size

Male patients usually received larger stentless prostheses than female patients: valve size 23 and 25 was implanted in 73% of the male patients and valve size 21 and 23 in 86% of the female patients.

The linear regression model revealed subcoronary implantation technique (p < 0.001), female gender (p < 0.001, body height (p < 0.001), age (p = 0.02), BMI (p = 0.046), re-replacement of the aortic valve (p = 0.01) as predictors for valve size.

### Determinants of mortality and survival time

Predictors of 30-day, 6-month mortality and survival time are presented in table 1. In a first step we examined whether valve size was an independent risk factor for outcome. To adjust for patient-related factors correlated with valve-size, we analysed the interactions of the predictors of valve size (see above) together with Body Surface Area (BSA) for all models. Continuous variables were checked for possible cut points: In the model of 30-day-mortality we studied the linearity of age for female and male patients separately. As a possible cut point for female patients may be over 70 years interaction terms such as female by age over 70, 71 etc. were successively included into the model. In the model of 6-months mortality BSA was checked for linearity. Since a possible cut point for BSA may be between 1.75 and 1.8 m2, BSA smaller than 1.75, 1.76, etc. were successively included into the model. In the Cox-model the survival curves were inspected for possible cut points at age of 79 or 80 years, body height at nearly 165 cm, BMI at 24 and for BSA at nearly 1.79. All interactions between these variables and variables of the linear regression models were analysed.

**Table 1:** Predictors of 30-day- and 6-months-mortality and survival time. Apparently valve size is a risk factor in the first step of the analysis. However, after adjustment for gender, age, BSA, BMI and body size, valve size disappeared (p < 0.05)

	**Models for**					
**Preoperative risk factors**	**30-day mortality**		**6-months mortality**		**Survival time**	
	**OR**	**95% CI**	**OR**	**95% CI**	**HR**	**95% CI**
Myocardial decompensation	2.9	1.1–7.2				
Diabetes mellitus			2.7	1.3–5.5	2.0	1.3–3.0
Atrial fibrillation			2.3	1.1–5.2	2.8	1.8–4.4
Peripheral vascular disease					2.2	1.2–4.0
Antithrombine III (%)	0.97	0.94–0.99	0.96	0.94–0.99		
Urea concentration (mg/dl)			1.02	1.01–1.03	1.02	1.011.02
Valve size	**p = 0.009**		**p = 0.05**		**p = 0.02**	

**After Variable transformation:**						
Female and age > 74 years	4.0	1.4–11.0				
BSA < 1.78 m^2^			3.0	1.51–6.07		
Female and age > 80 years					1.8	1.03–2.94
BMI < 24 and body size < 165 cm					1.7	1.02–2.93
**Valve size**	**p = 0.13**		**p = 0.47**		**p = 0.22**	

CI = Confidential Interval

HR = Hazard Ratio

OR = Odds Ratio

p = p-value of model improvement

After adjusting for these factors, valve size was no longer a risk factor in all 3 models (table 1), nor was indexed geometric orifice area.

### Determinants of QoL

The percentage of patients after AVR with NHP-scores higher than the age and gender matched general German population was 43% in the section energy, 25% in the section pain, 29% in the section emotional reaction, 27% for sleep, 18% for social isolation and 41% for physical mobility.

Preoperative risk factors for impaired QoL are shown in Table 2 (+coumarin). The model for impaired physical mobility had good discriminative powers (c-index = 0,78). C-indexes for energy, pain, emotional reaction, sleep, social isolation were 0,72, 0,37, 071, 071 and 0,74.

**Table 2:** Significant predictors of impaired QoL are listed

Risk factors	Energy [OR;95%CI]	Pain [OR;95%CI]	Emotional reaction [OR;95%CI]	Sleep [OR;95%CI]	Social isolation [OR;95%CI]	Physical mobility [OR;95%CI]
Age	1.1; 1.0–1.1	(> 76), 2.8; 1.7–4.9	(> 79), 2.6; 1.4–4.8	1.1; 05–1.2	(> 70), 5.6; 1.9–16.7),	1.1; 1.08–1.2
Female gender	2.2; 1.4–3.6			2.3; 1.4–3.9	2.9; 1.6–5.7	2.7; 1.6–4.3
Lower potassium ^1^	0.4; 0.2–0.7					
Higher creatinine ^2^	2.7; 1.3–5.7					
BMI ^3^		1.1; 1.0–1.2				1.0; 1.0–1.1
History of syncope		2.1; 1.0–4.1				
Advanced NYHAclass		2.3; 1.4–4.0	2.0; 1.2–3.3			1.7; 1.1–2.8
Lower hemoglobine		(< 13) 2.0; 1.2–3.5				
History of MI ^4^			3.2; 1.3–7.5			
COPD ^5^			2.6; 1.3–5.3			
Previous CABG			6.2; 4.5–7.9			
De Ritis-ratio (AST/ALT) ^6^			1.6; 1.1–2.4			
Neurological disorders			2.2; 1.0–4.8		3.7; 1.5–8.7	
Concomitant CABG				1.7; 1.01–2.8		
Higher urea^7^					1.01; 1.0–1.04	
Non-elective procedure						2.5; 1.4–4.7
Pacemaker before AVR						10.5; 1.1–99.6
Mean gradients (1 mmHg)						1.1; 1.0–1.1

Abbrevations: ^1^: potassium concentration (mmol/l); lower potassium probably as marker for diuretic therapy, ^2^: creatinine concentration (mg/dl), ^3^: body mass index, ^4^: Myocardial infarction, ^5^: Chronic obstructive pulmonary disease, ^6 ^De ritis ratio: De ritis-ratio as marker for liver damage, e.g. ethanol intake ^7 ^Urea concentration (mg/dl),

Including the size of implanted valves and the indexed geometric orifice area did not improve the models significantly (p < 0.1).

In order to assess the effect of missing data in the models for Quality of Life, we have recalculated all models for the worst and best case scenarios, where all missing data were imputed as "impaired QoL" respectively "non impaired QoL". In both, the worst- and best case scenarios, the identified risk factors for Quality of Life did not change and the c-indexes for all models decreased, suggesting that firstly, the models have enough stability and secondly, that the non responding patients, have a mixed risk profile, not exclusively belonging to the high risk or low risk group.

16% of the patients (N = 58) took coumarin during follow-up, mainly due to atrial fibrillation.

### Impact of early postoperative gradient on mortality and QoL

The mean and maximum transvalvular pressure gradients at hospital discharge for each valve size and different implantation technique are shown in table 3. Postoperative mean and maximum transvalvular pressure gradients did not influence 6-months mortality and survival times (table 1, p = 0.87, p = 0.82; respectively; p = 0.78, p = 0.9).

**Table 3:** Mean and maximum transvalvular pressure gradients (mmHg) at discharge (5 to 7 days after AVR)

	Subcoronary implantation				Total root replacement			
Valve size	Mean	N	Max	N	Mean	N	Max	N

19	29.8 ± 14.4	9	54.2 ± 23.2	9	-	0	-	0
21	22.0 ± 8.9	127	40.4 ± 14.5	119	13.7 ± 4.2	3	25.7 ± 7.5	3
23	19.6 ± 7.6	176	35.5 ± 12.8	149	13.5 ± 6.0	27	25.3 ± 10.1	26
25	17.0 ± 6.9	100	31.0 ± 14.9	80	10.8 ± 5.0	20	19.8 ± 10.9	19
27	14.8 ± 5.7	50	26.7 ± 9.4	47	7.2 ± 2.0	9	13.2 ± 4.3	9

The postoperative mean and maximum transvalvular pressure gradients had no impact on the QoL sections energy (p = 0.54, p = 0.33), pain (p = 0.34, p = 0.19), emotional reaction (p = 0.33, p = 0.08), sleep (p = 0.9, p = 0.89) and social isolation (p = 0.71, p = 0.81, respectively for mean and maximum pressure gradients). However, increasing postoperative mean transvalvular pressure gradients were identified as independent risk factors for impaired physical mobility (p = 0.002, Table 2).

In a logistic regression model for non-responding the postoperative mean and maximum transvalvular pressure gradients were no risk factors (p-values for model improvement: 0.69 and 0.42, respectively).

### Risk factors for higher transprosthetic gradients and influence of the surgeons

**Figure 3:**
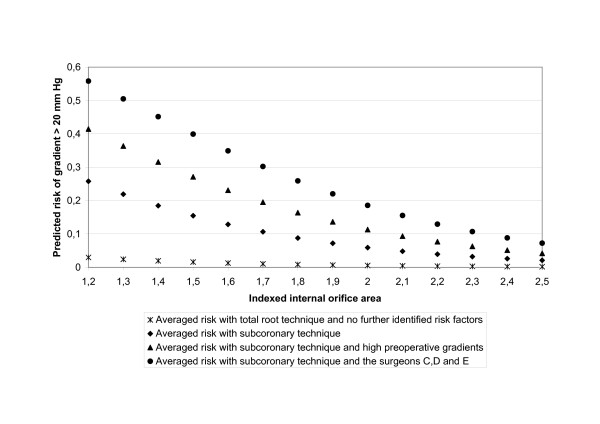
**Risk factors for higher transvalvular gradients after stentless valve implantation**. By solving the multivariate equations we had illuminated the importance of the dominant risk factors for higher gradients after stentless valve implantation: valve size in relation to BSA (IGOE), subcoronary technique, preoperative gradients, and the surgeons C, D, E. Other risk factors (cardiac, haematological) were less important (se table 4). With total root technique virtually no increase of the risk for higher gradients with decreasing IGOA was observed, whereas with subcoronary technique the risk increases exponentially.

As impaired physical mobility due to high transvalvular pressure gradients may reflect the impact of valve design on outcome and may in the consequence also affect mid-term survival, we were interested in the factors determining high early postoperative transvalvular gradients. As a dichotomic variable "mean gradient higher than 20 mm Hg yes or no" became significant (p = 0.008) in the model of impaired physical mobility, a saturated propensity score predicting mean transvalvular gradients higher than 20 mm Hg (table 4) was calculated. This score well describes patients with gradients higher than 20 (28% of all patients) as the c-index was 0.79 and the p-value of the variable "mean gradient higher than 20 mm Hg yes or no" became not significant (p = 0.057) after including the propensity score into the model of impaired physical mobility. Figure 3 shows the impact of several predicting factors on the risk of having a postoperative transvalvular pressure gradient higher than 20 mm Hg. The main factors predicting high postoperative pressure gradients are indexed geometric orifice area, subcoronary implantation technique, preoperative transvalvular gradients and the individual surgeon. Three surgeons (surgeons C-E) had significantly higher gradients than surgeons A, B, F and the mixed group M. Common parameters assessing "surgical experience" like years in cardiac surgery, number of valve cases performed or number of valves implanted did here not explain the differences between the surgeons concerning transvalvular gradients (table 5). These differences were observed in subcoronary technique for each valve size from 21 to 25 (table 6).

**Table 4:** Saturated propensity score predicting a mean transvalvular gradient after AVR > 20 mmHg

Predictors	Odds ratio	95% CI
Male gender	1.83	1–3.36
Body height (cm)	0.98	0.93–0.99
Subcoronary implantation technique	11.4	2.59–49.9
Indexed geometric orifice area	0.12	0.05–0.28
Less-experienced surgeon	3.64	2.34–5–66
Ejection fraction (%?)	1.01	1–1.03
Preoperative maximum transvalvular gradient (mmHg)	1.01	0.99–1.02
White blood cell count (cells*1000/μl)	1.11	1.02–1.22
Total serum protein (g/dl)	0.55	0.4–0.77
Glutamic-oxalacetic transaminase (U/l)	0.97	0.93–0.99
Potassium (mmol/l)	0.48	0.29–0.78
Mean corpuscular volume (fl)	1.04	0.99–1.08
Atrial fibrillation	0.6	0.3–1.2
Chronic pulmonary disease	1.5	0.81–2.75
Renal insufficiency	1.41	0.82–2.41
Peripheral occlusive arterial disease	0.56	0.2–1.56

CI – confidential interval

**Table 5:** Experience of the surgeons at end of 2004

**Surgeons**	Years in Cardiac Surgery	Major cardiac surgery performed [N]	Valve implantation performed [N]	Freestyle valve implantation performed [N]	Mean transprosthetic gradients in subcoronary technique (ANOVA, p < 0.001)
F	> 20	> 5000	> 1000	26	14
E	> 10	2500	400	66	17
C	> 20	> 4000	> 1000	280	18
A	> 10	2000	400	53	22
B	> 15	2500	500	74	22
D	> 10	1000	250	41	24

**Table 6:** Mean transvalvular gradients for the main valve sizes by surgeon

		Valve Size					
		21 mm		23 mm		25 mm	
Surgeons	N cases	*Mean *± *StDev [mmHg]*	*Range [mmhg]*	*Mean *± *StDev [mmHg]*	*Range [mmHg]*	*Mean *± *StDev [mmHg]*	*Range [mmHg]*

Trainees	43	19 ± 2.4	0	16 ± 1.8	.-	17 ± 2.3	.-
A	53	24 ± 2.3	20	22 ± 1.7	24	16 ± 2.3	22
B	74	25 ± 1.9	27	21 ± 1.3	35	19 ± 1.8	28
C	280	21 ± 1.1	40	18 ± 0.8	27	16 ± 0.9	28,4
D	41	23 ± 2.7	38	26 ± 1.7	35	19 ± 2.8	35
E	67	20 ± 1.9	25	17 ± 1.4	30	13 ± 1.7	16
F	28	10 ± 8.2	27	12 ± 2.0	19	12 ± 2.6	18

ANOVA		P = 0.1		p < 0.001		P = 0.1	

**Figure 4:**
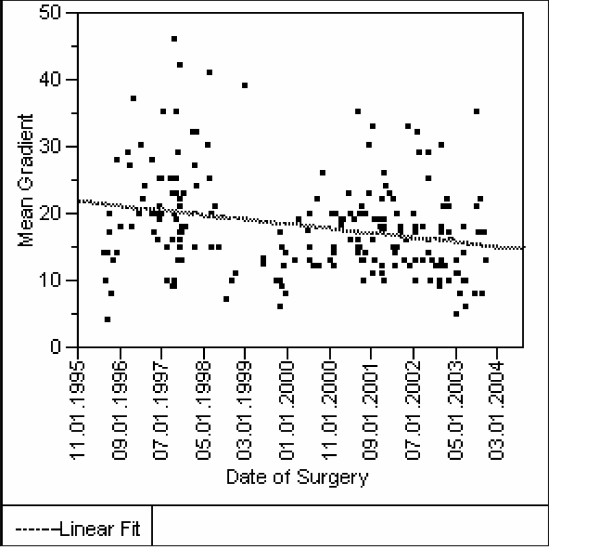
**Learning curve of one surgeon concerning transprosthetic gradients after stentless valve implantation [p < 0.001]**. The example of one surgeon shows that the mean gradients in subcoronary technique decrease during a time period of 8 years. Beside proper valve sizing and use of total root technique in difficult cases with small aortic roots, the phenomena can be best explained by the increasing ability of the surgeon to fit the valve smoothly into the aortic root and to handle the various aortic root geometries.

We observed learning curves in surgeons A, C, D, E, whereas gradients through stentless valves implanted by surgeons B, F and the mixed group M were low already in the beginning of their experience. An example of a learning curve for one surgeon is provided in figure 4.

## Discussion

Encouraged by previous reports about the excellent hemodynamic performance and the good initial experience, stentless valves are frequently implanted by all surgeons in our institution.

The 30 day mortality in our study equals to the reports of a multicenter evaluation of the Freestyle valve (3.1% versus 3.0%) [18] and the survival rate, after 5 years was similar to a series with a comparable mean age of 75 years [19] (73%, versus 72% in our study). In accordance with other studies we observed virtually no structural valve deterioration or important aortic insufficiency with the Freestyle stentless valves [3].

Nevertheless, we observed in a considerable percentage of patients higher transvalvular gradients. They were in average higher than reported previously [1,1,3,11,20,21], what may be partly explained by the more frequent use of subcoronary technique in patients with small aortic roots., but also previous studies reported a heterogeneity of the transvalvular gradients and suboptimal hemodynamics with the freestyle stentless valve early postoperative [1,12,13,22]. Up to now, both the risk factors for higher gradients and their clinical relevance are still poorly defined. It was argued that echocardiography tends to overestimate the transvalvular gradient in stentless valves, because the velocity profile of stentless valves is more parabolic in the early postoperative period, [1] and furthermore that elevated gradients will decline by resolution of paravalvular haematoma and the normalization of postoperative hemodynamics. [1,3,12]. On the other hand, previous studies showed that transvalvular gradients remained high at follow-up and left ventricular mass regression was subsequently less [12,22]. Up till now, a possible association of postoperative gradients with clinical symptoms was not studied in stentless valves. Even, in other valve types, the clinical significance of residual left ventricular outflow tract obstruction after AVR is still under debate [23].

We could not detect an impact of neither the early postoperative transvalvular gradient nor the IGOA on morbidity or mid-term mortality, but physical mobility at follow-up was impaired with increasing mean transvalvular gradient especially with mean gradients > 20 mmHg. Also our finding that apart from physical mobility, none of the other NHP sections: energy, pain, emotional reaction, sleep, or social isolation were affected, indicates an exercise dependent mechanism similar to what occurs in patients with hemodynamically important stenosis. Previous studies have suggested that the residual transprosthetic pressure gradient results in an increased LV workload, thus hampering the regression of LV mass after AVR and subsequently functional recovery after AVR [24,26]. In addition it was demonstrated that, in patients with AS and angiographically normal coronary arteries, the improvement of coronary flow reserve after AVR is directly dependent on the improvement of valve EOA that is achieved with AVR [27]. Hence, the increased LV systolic pressure associated with residual transvalvular gradients may compromise the normalization of coronary flow reserve after AVR. Both mechanisms predispose to decreased exercise tolerance and explain our finding of an impaired physical mobility in patients with higher gradients after AVR.

In a recent study no risk-adjusted impact of mean transvalvular gradients or IGOA on functional health-related QoL was detected after AVR with mainly stented valves [28]. The Duke Activity Status Index (DASI), used in this study to measure activities of daily living such as household tasks, ambulation or personal care, shows similarities to the section physical mobility of the NHP. Nevertheless, this study was different from ours regarding the study design. It may be possible that, due to the fact that the mean follow-up time in our study was more than 3 times longer than in the above mentioned study, we were able to prove an impact of the postoperative gradient on QoL.

According to our data in stentless valves higher gradients depend beyond the physical valve sizes and its relation to body size, mainly on the interaction of subcoronary technique, the preoperative gradients with the individual surgeons.

By solving the multivariate equations in figure 2 we had illuminated how with total root technique virtually no increase of the risk for higher gradients with decreasing IGOA was observed, whereas with subcoronary technique the risk increase exponentially. Thus previous reports could be redefined, where a threshold of a valve size equal or smaller than 23 was mentioned as risk factor for higher gradients [1,12]. In contrary to a study about the outcome after human tissue valves for aortic valve replacement, a limited performance of the individual surgeon in subcoronary technique did not result in more initial aortic regurgitation and early reoperation our series. [29]

It was already assumed that the performance of stentless valves depends to a larger extent on the surgical experience [1,12]. Up to now only data were available concerning 30 day mortality, where a significant decrease with operator experience for both subcoronary and total root technique was described [18]. In our study no significant effect of individual surgeons on mortality was detectable, but we were able to show to which degree the hemodynamic performance of stentless valves depends on the individual surgeon and proved its clinical consequences. In contrary to the study about learning curve of tissue valve implantation, where an experience of more than 38 cases was associated with better performance, we could not define the number of cases needed, until the constantly better gradients where achieved, due to large variations between the surgeons; their skills and differences in their individual histories of training seems to be more important than experience over time and the number of cases.

According to our and others experiences [1,12] higher transvalvular gradients through stentless valves may develop by already slight distortions of the valve, horizontal or vertical folding of valve tissue into the outflow tract, oversizing, impaired movements of the non-coronary cusp or due to paravalvular hematoma. Especially in patients with a discrepancy between annulus and sinotubular junction dimensions, calcification in the aortic sinuses, unusual angles between the coronary ostia, bicuspid valves or small aortic roots, the difficulty of stentless valves implantation increases whereas the reproducibility decreases.

### Limitation

In our study we considered both transvalvular gradients and IGOA as possible determinant of outcome, but the Effective Orifice Area (EOAs) was not assessed. Nevertheless a correlation of the mean transvalvular gradients with the EOA, the impedance to the left ventricular outflow and left ventricular mass regression is well established for native valves, conventional prosthesis [30] and stentless xenografts, in rest and in exercise [1,3,8,12,13,31]. Despite its known limitations, an accurate measurement of the pressure gradient is still sufficient to make clinical decision [30]. In order to adjust for remaining discrepancies between transvalvular gradient and EOAs, we adjusted for ejection fraction and other variables known to influence the gradients like blood composition, ejection fraction, atrial fibrillation and preoperative gradients.

## Conclusion

The implantation of Freestyle stentless valves in subcoronary technique is technically demanding and therefore affords an extraordinary expertise, especially in managing small valve sizes (in relation to BSA), cases with a difficult aortic root geometry, sinus calcifications and severe left ventricular hypertrophy. Transvalvular gradients rise easily when the valve does not smoothly fit within the aortic root (whereas aortic insufficiency is extremely uncommon). Despite the well-known limitations of the transvalvular gradient for measuring performance of aortic valves, in the majority of patients it reflected sufficiently the technical quality of implantation and correlated significantly with physical mobility years after the operation. In order to avoid unfavourable performance curves ("learning curves") in technically demanding procedures like stentless valve implantation it is recommendable to apply more profoundly the recognized methods of surgical teaching (e.g. prolonged expert guidance, cognitive task analysis, model training [32]) and thereby optimizing the diffusion of such a new technology [33].

## Competing interests

One author, Ines Florath, PhD: Research Fellow – Department of Thoracic and Cardiovascular Surgery, Heart Institute Lahr/Baden, Germany, get grants from Medtronic Inc., but the company has not been involved in any aspect of data collection or analysis, writing or reading the manuscript.

Alexander Albert, MD: Consultant of Thoracic and Cardiovascular Surgery, Heart Institute Lahr/Baden, Germany : 'The author declare that he has no competing interests'.

Ulrich Rosendahl, MD: Consultant of Thoracic and Cardiovascular Surgery Department, Heart Institute Lahr/Baden, Germany: 'The author declare that he has no competing interests'

Wael Hassanein, MD: Resident of Thoracic and Cardiovascular Surgery, Heart Institute Lahr/Baden, Germany: 'The author declare that he has no competing interests'

Eberhard v. Hodenberg, MD, PhD: Head of Cardiology Department, Heart Institute Lahr/Baden, Germany: 'The author declare that he has no competing interests'

Stefan Bauer, MD: Consultant of Thoracic and Cardiovascular Surgery, Heart Institute Lahr/Baden, Germany: 'The author declare that he has no competing interests'

Ina Carolin Ennker, MD: Consultant of Thoracic and Cardiovascular Surgery, Heart Institute Lahr/Baden, Germany: 'The author declare that she has no competing interests'

Jürgen Ennker, MD: Head of Thoracic and Cardiovascular Surgery Department, Heart Institute Lahr/Baden, Germany: 'The author declare that he has no competing interests'

## Authors' contributions

Alexander Albert established the database, helped with collection and interpretation of the data, designed the study and drafted the manuscript

Ines Florath performed the follow-up studies and statistical analysis and helped with interpretation of data

Ulrich Rosendahl helped with collection and interpretation of data

Wael Hassanein helped with interpretation of data and revised the manuscript critically

Eberhard v. Hodenberg was responsible for perioperative echocardiography, helped with interpretation of data and revised the manuscript critically

Stefan Bauer helped with acquisition of data

Ina Carolin Ennker helped with acquisition of data

Jürgen Ennker helped with acquisition and interpretation of data

All authors read and approved the final manuscript.

## Supplementary Material

Additional file 1 Distribution of age. The figure illustrates the age distribution of our study population Click here for file

Additional file 2 Patient's and procedural variability. The data provided represent a list of risk-adjusting variables (without the laboratory values, see additional file 2) Click here for file

Additional file 3 Laboratory values. The data provided represent laboratory values used for risk-adjustment Click here for file

Additional file 4 Data of patients requiring reoperation after implantation of Freestyle^® ^stentless bioprostheses. The data provided describe the pathology of those cases where after implantation of Freestyle^® ^stentless bioprostheses a reoperation was necessary Click here for file
