# Supplementary figures and images for: Effect of surgeon on transprosthetic gradients after aortic valve replacement with Freestyle® stentless bioprosthesis and its consequences: A follow-up study in 587 patients
Source: J Cardiothorac Surg. 2007 Oct 5;2:40. doi: 10.1186/1749-8090-2-40 (PMC2146998; doi:10.1186/1749-8090-2-40)

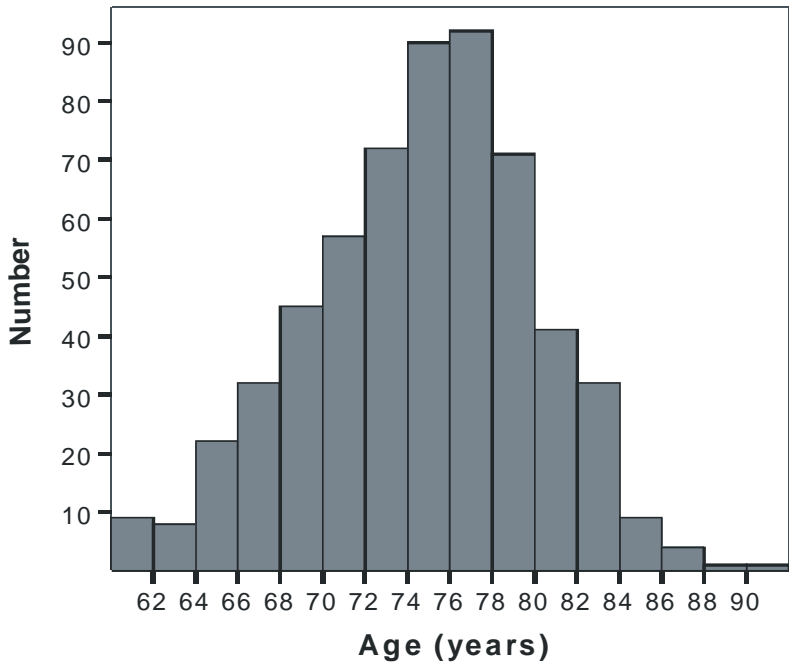

Supplement: Additional file 1 — Distribution of age. The figure illustrates the age distribution of our study population [file 1749-8090-2-40-S1.pdf]
