# Supplementary material for: Effect of surgeon on transprosthetic gradients after aortic valve replacement with Freestyle® stentless bioprosthesis and its consequences: A follow-up study in 587 patients
Source: J Cardiothorac Surg. 2007 Oct 5;2:40. doi: 10.1186/1749-8090-2-40 (PMC2146998; doi:10.1186/1749-8090-2-40)
Supplement: Additional file 2 — Patient's and procedural variability. The data provided represent a list of risk-adjusting variables (without the laboratory values, see additional file 2) [file 1749-8090-2-40-S2.doc]

#### Additional file Table 1 - Patient’s and procedural variability

| N | 587 | unknown |
| --- | --- | --- |
| Female | 309 (53) | 0 |
| Age (years) | 75±5 | 0 |
| BSA (m^2^) | 1.84±0.2 | 0 |
| BMI (kg/m^2^) | 26.5±4.3 | 0 |
| Coronary artery bypass grafting | 227 (39) | 0 |
| Previous open heart surgery | 16 (2.7) | 1 |
| Emergency procedure | 4 (0.7) | 0 |
| Aortic root replacement | 69 (11.8) | 0 |
| Mitral valve replacement | 3 (0.5) | 0 |
| Previous myocardial infarction | 49 (8) | 0 |
| Diabetes mellitus | 130 (22) | 1 |
| History of congestive heart failure | 114 (19) | 2 |
| Hypertension | 339 (58) | 2 |
| Hyperlipidaemia | 249 (42) | 2 |
| Syncope | 86 (15) | 1 |
| History of venous or arterial embolism | 14 (2) | 1 |
| Renal disease | 97 (16) | 0 |
| Peripheral occlusive arterial disease | 31 (5) | 2 |
| Chronic pulmonary disease | 73 (12) | 0 |
| Infectious disease | 11 (2) | 1 |
| Atrial fibrillation | 67 (11) | 0 |
| Heart pacemaker | 110(2) | 0 |
| Neurological disorders (affecting ambulation or day-to-day functioning) | 59 (10) | 2 |
| NYHA class |  | 5 |
| I | 26 (4) |  |
| II | 237 (41) |  |
| III | 264 (45) |  |
| IV | 56 (10) |  |
