# Supplementary material for: Effect of surgeon on transprosthetic gradients after aortic valve replacement with Freestyle® stentless bioprosthesis and its consequences: A follow-up study in 587 patients
Source: J Cardiothorac Surg. 2007 Oct 5;2:40. doi: 10.1186/1749-8090-2-40 (PMC2146998; doi:10.1186/1749-8090-2-40)
Supplement: Additional file 3 — Laboratory values. The data provided represent laboratory values used for risk-adjustment [file 1749-8090-2-40-S3.doc]

#### Additional file Table 2 - Laboratory values

mean±SD unknown (%)

Red blood cell count (cells*1000/µl) 4.32±0.57 22(3.7)

White blood cell count (cells*1000/µl) 7.5±4.1 22(3.7)

Platelet count (cells*1000/µl) 227±77 22(3.7)

Hemoglobin (g/dl) 13±2 22(3.7)

Mean corpuscular haemoglobin concentration (%) 33.4±1.2? 22(3.7)

Mean corpuscular hemoglobin (pg) 30.6±2.2 22(3.7)

Mean corpuscular volume (fl) 92±5 22(3.7)

Hematocrit (%) 39±5 22(3.7)

Billirubin (mg/dl) 0.61±0.34 26(4.4)

Glucose (mg/dl) 129±47 22(3.7)

Cholesterol (mg/dl) 211±46 25(4.2)

Creatinine (mg/dl) 1.1±0.4 21(3.6)

Urea (mg/dl) 47±23 21(3.6)

Total serumprotein (g/dl) 7.6±0.6 37(6.3)

Antithrombin III (%) 93.4±1.6 34(5.8)

Partial thromboplastine time (sec) 37±10 18(3.1)

International normalisatino ratio (INR) 1.1±0.1 20(3.4)

Glutamic-oxalacetic transaminase (U/l) 12.1±7.0 21(3.6)

Lactate dehydrogenase (U/l) 193±52 21(3.6)

Glutamic-pyruvic transaminase (U/l) 13±12 27(4.6)

Creatininekinase (U/l) 36.9±34.3 21(3.6)

Tyroid stimulating hormon (mU/l) 1.0±1.6 43(7.3)

Sodium (mmol/l) 140±3 21(3.6)

Potassium (mmol/l) 4.2±0.4 21(3.6)
