# Supplementary material for: Effect of surgeon on transprosthetic gradients after aortic valve replacement with Freestyle® stentless bioprosthesis and its consequences: A follow-up study in 587 patients
Source: J Cardiothorac Surg. 2007 Oct 5;2:40. doi: 10.1186/1749-8090-2-40 (PMC2146998; doi:10.1186/1749-8090-2-40)
Supplement: Additional file 4 — Data of patients requiring reoperation after implantation of Freestyle® stentless bioprostheses. The data provided describe the pathology of those cases where after implantation of Freestyle® stentless bioprostheses a reoperation was necessary [file 1749-8090-2-40-S4.doc]

#### Additional file Table 3 Data of patients requiring reoperation after implantation of Freestyle® stentless bioprostheses

|  | Subcoronary | Root replacement |
| --- | --- | --- |
| Re-replacement (all)  Non-structural dysfunction  Operated valvular endocarditis  unknown | 10  7  2  1 | 2  0  2 |
| Time of reoperation after primary AVR  Non-structural dysfunction  Operated valvular endocarditis | 1.3±1.6 months  3.4±2.4 months | 9.2±3.9 months |
| Type of re-replaced valve  Non-structural dysfunction    Operated valvular endocarditis | SJM R(N=3), CM(N=2), Leak Closure (N=2)  Mos(N=1), MH(N=1) | FreeTRR(N=1), TA(N=1) |
| Mortality within 2 months | 4 (40%) | 2 (100%) |
| Mean follow-up time of survivors | 21.6±15months |  |

SJM R -SJM Regent –, CM-Carbomedics, Mos - Medtronic Mosaic, MH - Medtronic Hall, FreeTRR -Freestyle TRR, TA - Tissuemed Aortic Root, LC - Closure of paravalvular leak,
